# Supplementary figures and images for: Network Architecture and Mutational Sensitivity of the C. elegans Metabolome
Source: Front Mol Biosci. 2018 Jul 31;5:69. doi: 10.3389/fmolb.2018.00069 (PMC6079199; doi:10.3389/fmolb.2018.00069)

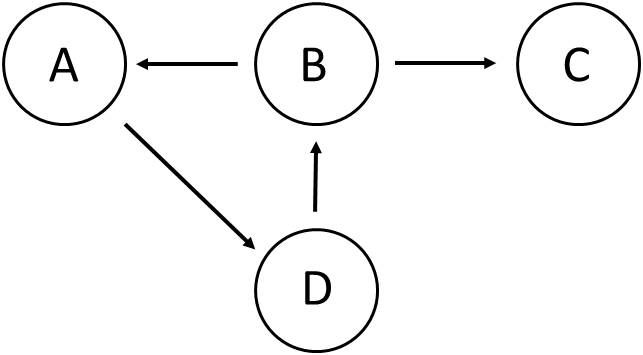

Supplement: Supplementary Figure 1 — Depiction of shortest path length in a directed network. [file Image_1.TIF]

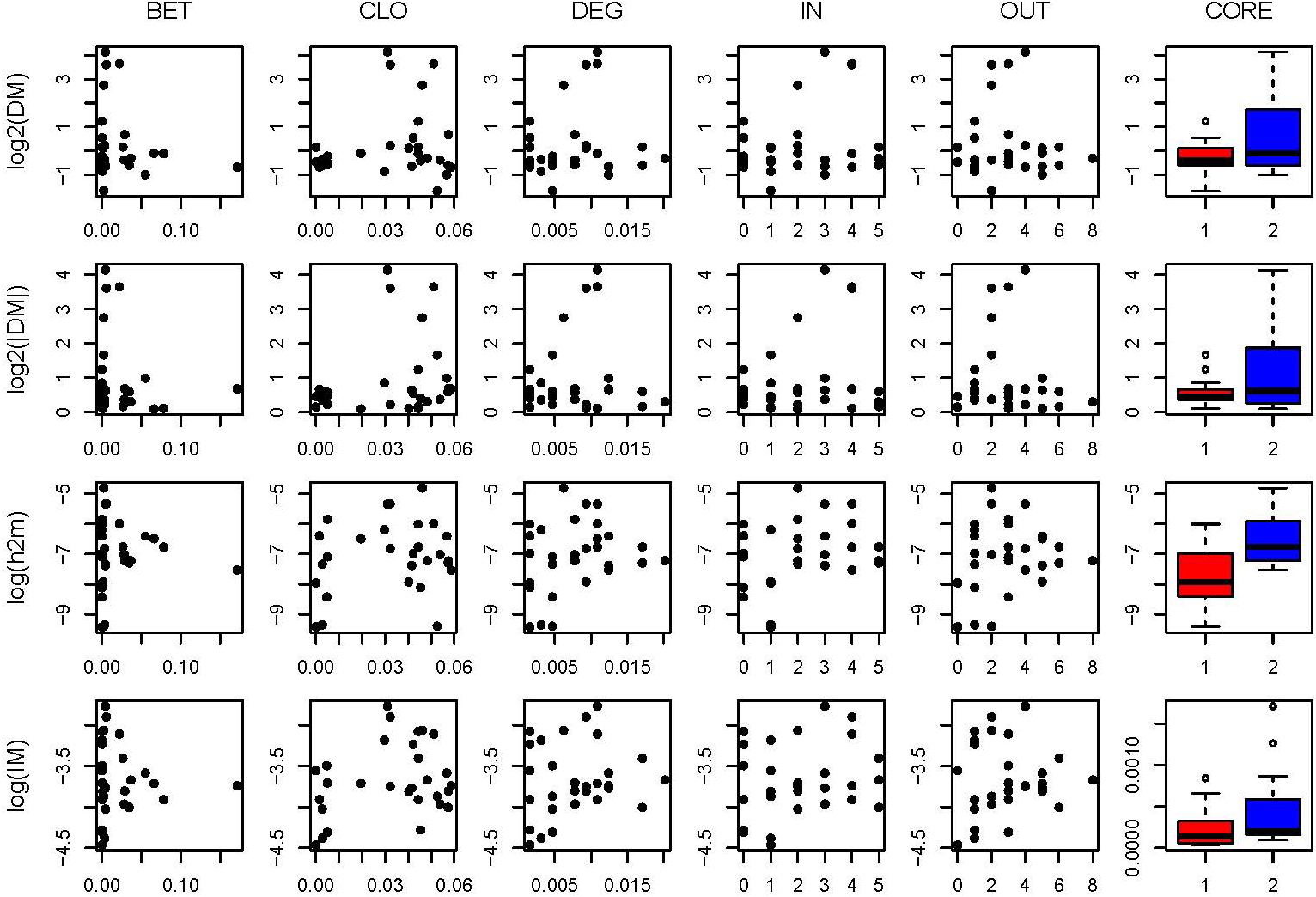

Supplement: Supplementary Figure 2 — Plots of mutational parameters vs. network statistics. [file Image_2.TIF]

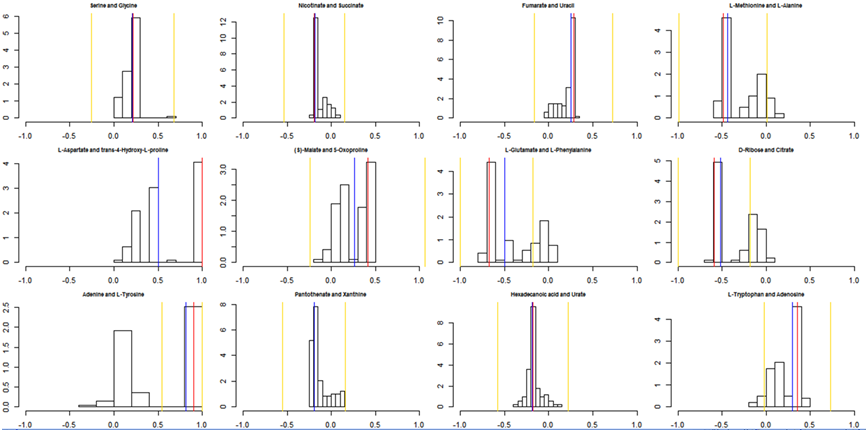

Supplement: Supplementary Figure 3 — Bootstrap distributions of rM with six randomly chosen covariates. [file Image_3.TIF]

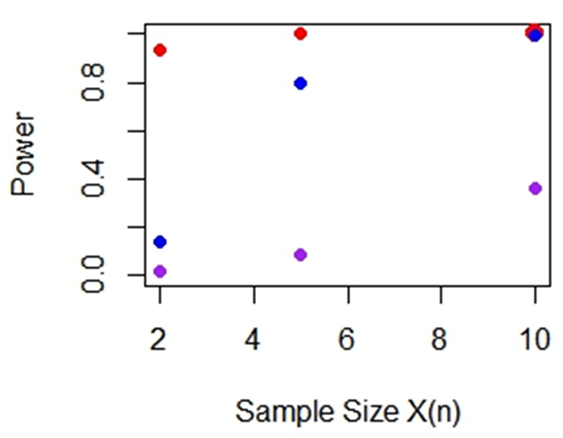

Supplement: Supplementary Figure 4 — Power curve for metabolite sample size. [file Image_4.TIF]
