# Supplementary material for: Network Architecture and Mutational Sensitivity of the C. elegans Metabolome
Source: Front Mol Biosci. 2018 Jul 31;5:69. doi: 10.3389/fmolb.2018.00069 (PMC6079199; doi:10.3389/fmolb.2018.00069)
Supplement: Supplementary Table 3 — Canonical correlation analysis. [file Table_3.DOCX]

**Supplementary Table S3**. Canonical correlation analysis, output from SAS Proc CANCORR. Variable abbreviations are as in Table 2 in the main text.

Normal Quantile-Quantile Plot

The CANCORR Procedure

| **Network stats** | 6 |
| --- | --- |
| **Mutation stats** | 4 |
| **Observations** | 29 |

| **Means and Standard Deviations** | | |
| --- | --- | --- |
| **Variable** | **Mean** | **Standard Deviation** |
| **BET** | 0.019989 | 0.036299 |
| **CLO** | 0.032784 | 0.020934 |
| **DEG** | 0.007818 | 0.005191 |
| **INDEG** | 2.103448 | 1.654967 |
| **OUTDEG** | 2.931034 | 1.998768 |
| **CORE** | 1.551724 | 0.506120 |
| **log2(ΔM)** | 0.248730 | 1.457899 |
| **log2\|ΔM\|** | 0.942483 | 1.126741 |
| **log(h2M)** | -7.061328 | 1.169426 |
| **log(IM)** | -8.452516 | 1.016196 |

| Normal Quantile-Quantile Plot |
| --- |

The CANCORR Procedure

Correlations Among the Original Variables

| **Correlations Among the Network stats** | | | | | | |
| --- | --- | --- | --- | --- | --- | --- |
|  | **BET** | **CLO** | **DEG** | **INDEG** | **OUTDEG** | **CORE** |
| **BET** | 1.0000 | 0.4270 | 0.4886 | 0.5197 | 0.3869 | 0.4834 |
| **CLO** | 0.4270 | 1.0000 | 0.5223 | 0.5108 | 0.4506 | 0.5224 |
| **DEG** | 0.4886 | 0.5223 | 1.0000 | 0.8966 | 0.9303 | 0.7904 |
| **INDEG** | 0.5197 | 0.5108 | 0.8966 | 1.0000 | 0.6716 | 0.8248 |
| **OUTDEG** | 0.3869 | 0.4506 | 0.9303 | 0.6716 | 1.0000 | 0.6391 |
| **CORE** | 0.4834 | 0.5224 | 0.7904 | 0.8248 | 0.6391 | 1.0000 |

| **Correlations Among the Mutation stats** | | | | |
| --- | --- | --- | --- | --- |
|  | **log2(ΔM)** | **log2\|ΔM\|** | **log(h2M)** | **log(IM)** |
| **log2(ΔM)** | 1.0000 | 0.8430 | 0.6229 | 0.7096 |
| **log2\|ΔM\|** | 0.8430 | 1.0000 | 0.5305 | 0.6855 |
| **log(h2M)** | 0.6229 | 0.5305 | 1.0000 | 0.7219 |
| **log(IM)** | 0.7096 | 0.6855 | 0.7219 | 1.0000 |

| **Correlations Between the Network stats and the Mutation stats** | | | | |
| --- | --- | --- | --- | --- |
|  | **log2(ΔM)** | **log2\|ΔM\|** | **log(h2M)** | **log(IM)** |
| **BET** | -0.1573 | -0.1373 | 0.0315 | -0.0640 |
| **CLO** | 0.1382 | 0.2088 | 0.2144 | 0.2753 |
| **DEG** | 0.0882 | 0.0640 | 0.2486 | 0.1534 |
| **INDEG** | 0.2231 | 0.2291 | 0.2998 | 0.2071 |
| **OUTDEG** | -0.0372 | -0.0826 | 0.1675 | 0.0851 |
| **CORE** | 0.3324 | 0.2823 | 0.5297 | 0.2994 |

| Normal Quantile-Quantile Plot |
| --- |

The CANCORR Procedure

Canonical Correlation Analysis

| Note: | The correlation matrix for the Network stats is less than full rank.  Therefore, some canonical coefficients will be zero. |
| --- | --- |

|  | **Canonical Correlation** | **Adjusted Canonical Correlation** | **Approximate Standard Error** | **Squared Canonical Correlation** | **Eigenvalues of Inv(E)*H = CanRsq/(1-CanRsq)** | | | | **Test of H0: The canonical correlations in the current row and all that follow are zero** | | | | |
| --- | --- | --- | --- | --- | --- | --- | --- | --- | --- | --- | --- | --- | --- |
|  |  |  |  |  | **Eigenvalue** | **Difference** | **Proportion** | **Cumulative** | **Likelihood Ratio** | **Approximate F Value** | **Num DF** | **Den DF** | **Pr > F** |
| **1** | 0.691021 | 0.588106 | 0.098741 | 0.477511 | 0.9139 | 0.6424 | 0.7074 | 0.7074 | 0.37080209 | 1.17 | 20 | 67.282 | 0.3045 |
| **2** | 0.462070 | 0.308642 | 0.148633 | 0.213508 | 0.2715 | 0.1835 | 0.2101 | 0.9175 | 0.70968352 | 0.64 | 12 | 55.852 | 0.7954 |
| **3** | 0.284422 | 0.152769 | 0.173694 | 0.080896 | 0.0880 | 0.0694 | 0.0681 | 0.9856 | 0.90234087 | 0.39 | 6 | 44 | 0.8836 |
| **4** | 0.135050 | 0.101524 | 0.185535 | 0.018239 | 0.0186 |  | 0.0144 | 1.0000 | 0.98176139 | 0.21 | 2 | 23 | 0.8092 |

| **Multivariate Statistics and F Approximations** | | | | | |
| --- | --- | --- | --- | --- | --- |
| **S=4 M=0 N=9** | | | | | |
| **Statistic** | **Value** | **F Value** | **Num DF** | **Den DF** | **Pr > F** |
| **Wilks' Lambda** | 0.37080209 | 1.17 | 20 | 67.282 | 0.3045 |
| **Pillai's Trace** | 0.79015359 | 1.13 | 20 | 92 | 0.3322 |
| **Hotelling-Lawley Trace** | 1.29197746 | 1.23 | 20 | 37.109 | 0.2860 |
| **Roy's Greatest Root** | 0.91391454 | 4.20 | 5 | 23 | 0.0074 |
| **NOTE: F Statistic for Roy's Greatest Root is an upper bound.** | | | | | |

| Normal Quantile-Quantile Plot |
| --- |

The CANCORR Procedure

Canonical Correlation Analysis

| **Raw Canonical Coefficients for the Network stats** | | | | |
| --- | --- | --- | --- | --- |
|  | **Network1** | **Network2** | **Network3** | **Network4** |
| **BET** | -12.49447713 | -16.17118383 | -1.567025296 | 25.933201835 |
| **CLO** | -1.526599565 | 30.783461734 | 46.533527523 | 4.8578941487 |
| **DEG** | -177.7998386 | -260.1861635 | 168.97440256 | -231.4122036 |
| **INDEG** | 0.1958530022 | 1.1417746945 | -0.578917885 | 0.5378344477 |
| **OUTDEG** | 0 | 0 | 0 | 0 |
| **CORE** | 2.8727010738 | -1.647218512 | -0.08787259 | -0.100365438 |

| **Raw Canonical Coefficients for the Mutation stats** | | | | |
| --- | --- | --- | --- | --- |
|  | **Mutation1** | **Mutation2** | **Mutation3** | **Mutation4** |
| **log2(ΔM)** | 0.2605189825 | -0.067736655 | -0.76232896 | -1.139859594 |
| **log2\|ΔM\|** | 0.2512287193 | 0.9267300964 | 0.082951394 | 1.4109503763 |
| **log(h2M)** | 0.7902976719 | -0.816191231 | 0.0489482886 | 0.5848878979 |
| **log(IM)** | -0.570164106 | 0.3609462863 | 1.345722301 | -0.743281593 |

| Normal Quantile-Quantile Plot |
| --- |

The CANCORR Procedure

Canonical Correlation Analysis

| **Standardized Canonical Coefficients for the Network stats** | | | | |
| --- | --- | --- | --- | --- |
|  | **Network1** | **Network2** | **Network3** | **Network4** |
| **BET** | -0.4535 | -0.5870 | -0.0569 | 0.9414 |
| **CLO** | -0.0320 | 0.6444 | 0.9741 | 0.1017 |
| **DEG** | -0.9230 | -1.3507 | 0.8772 | -1.2014 |
| **INDEG** | 0.3241 | 1.8896 | -0.9581 | 0.8901 |
| **OUTDEG** | 0.0000 | 0.0000 | 0.0000 | 0.0000 |
| **CORE** | 1.4539 | -0.8337 | -0.0445 | -0.0508 |

| **Standardized Canonical Coefficients for the Mutation stats** | | | | |
| --- | --- | --- | --- | --- |
|  | **Mutation1** | **Mutation2** | **Mutation3** | **Mutation4** |
| **log2(ΔM)** | 0.3798 | -0.0988 | -1.1114 | -1.6618 |
| **log2\|ΔM\|** | 0.2831 | 1.0442 | 0.0935 | 1.5898 |
| **log(h2M)** | 0.9242 | -0.9545 | 0.0572 | 0.6840 |
| **log(IM)** | -0.5794 | 0.3668 | 1.3675 | -0.7553 |

| Normal Quantile-Quantile Plot |
| --- |

The CANCORR Procedure

Canonical Structure

| **Correlations Between the Network stats and Their Canonical Variables** | | | | |
| --- | --- | --- | --- | --- |
|  | **Network1** | **Network2** | **Network3** | **Network4** |
| **BET** | -0.0469 | -0.3926 | 0.2682 | 0.8359 |
| **CLO** | 0.2175 | 0.2180 | 0.8953 | 0.3044 |
| **DEG** | 0.2785 | -0.2658 | 0.4640 | 0.0695 |
| **INDEG** | 0.4438 | 0.0151 | 0.2597 | 0.3123 |
| **OUTDEG** | 0.0984 | -0.4570 | 0.5611 | -0.1423 |
| **CORE** | 0.7558 | -0.2898 | 0.3401 | 0.2420 |

| **Correlations Between the Mutation stats and Their Canonical Variables** | | | | |
| --- | --- | --- | --- | --- |
|  | **Mutation1** | **Mutation2** | **Mutation3** | **Mutation4** |
| **log2(ΔM)** | 0.7829 | 0.4472 | -0.0266 | -0.4316 |
| **log2\|ΔM\|** | 0.6963 | 0.7060 | 0.1244 | 0.0340 |
| **log(h2M)** | 0.8926 | -0.1973 | 0.4018 | -0.0530 |
| **log(IM)** | 0.5514 | 0.3235 | 0.6843 | -0.3509 |

| **Correlations Between the Network stats and the Canonical Variables of the Mutation stats** | | | | |
| --- | --- | --- | --- | --- |
|  | **Mutation1** | **Mutation2** | **Mutation3** | **Mutation4** |
| **BET** | -0.0324 | -0.1814 | 0.0763 | 0.1129 |
| **CLO** | 0.1503 | 0.1007 | 0.2547 | 0.0411 |
| **DEG** | 0.1924 | -0.1228 | 0.1320 | 0.0094 |
| **INDEG** | 0.3067 | 0.0070 | 0.0739 | 0.0422 |
| **OUTDEG** | 0.0680 | -0.2112 | 0.1596 | -0.0192 |
| **CORE** | 0.5223 | -0.1339 | 0.0967 | 0.0327 |

| **Correlations Between the Mutation stats and the Canonical Variables of the Network stats** | | | | |
| --- | --- | --- | --- | --- |
|  | **Network1** | **Network2** | **Network3** | **Network4** |
| **log2(ΔM)** | 0.5410 | 0.2067 | -0.0076 | -0.0583 |
| **log2\|ΔM\|** | 0.4812 | 0.3262 | 0.0354 | 0.0046 |
| **log(h2M)** | 0.6168 | -0.0911 | 0.1143 | -0.0072 |
| **log(IM)** | 0.3810 | 0.1495 | 0.1946 | -0.0474 |

| Normal Quantile-Quantile Plot |
| --- |

The CANCORR Procedure

Canonical Redundancy Analysis

| **Raw Variance of the Network stats Explained by** | | | | | |
| --- | --- | --- | --- | --- | --- |
| **Canonical Variable Number** | **Their Own Canonical Variables** | | **Canonical R-Square** | **The Opposite Canonical Variables** | |
|  | **Proportion** | **Cumulative Proportion** |  | **Proportion** | **Cumulative Proportion** |
| **1** | 0.1036 | 0.1036 | 0.4775 | 0.0495 | 0.0495 |
| **2** | 0.1226 | 0.2262 | 0.2135 | 0.0262 | 0.0756 |
| **3** | 0.2106 | 0.4368 | 0.0809 | 0.0170 | 0.0927 |
| **4** | 0.0521 | 0.4889 | 0.0182 | 0.0009 | 0.0936 |

| **Raw Variance of the Mutation stats Explained by** | | | | | |
| --- | --- | --- | --- | --- | --- |
| **Canonical Variable Number** | **Their Own Canonical Variables** | | **Canonical R-Square** | **The Opposite Canonical Variables** | |
|  | **Proportion** | **Cumulative Proportion** |  | **Proportion** | **Cumulative Proportion** |
| **1** | 0.5732 | 0.5732 | 0.4775 | 0.2737 | 0.2737 |
| **2** | 0.2104 | 0.7836 | 0.2135 | 0.0449 | 0.3187 |
| **3** | 0.1252 | 0.9088 | 0.0809 | 0.0101 | 0.3288 |
| **4** | 0.0912 | 1.0000 | 0.0182 | 0.0017 | 0.3304 |

| Normal Quantile-Quantile Plot |
| --- |

The CANCORR Procedure

Canonical Redundancy Analysis

| **Standardized Variance of the Network stats Explained by** | | | | | |
| --- | --- | --- | --- | --- | --- |
| **Canonical Variable Number** | **Their Own Canonical Variables** | | **Canonical R-Square** | **The Opposite Canonical Variables** | |
|  | **Proportion** | **Cumulative Proportion** |  | **Proportion** | **Cumulative Proportion** |
| **1** | 0.1508 | 0.1508 | 0.4775 | 0.0720 | 0.0720 |
| **2** | 0.0942 | 0.2451 | 0.2135 | 0.0201 | 0.0921 |
| **3** | 0.2645 | 0.5095 | 0.0809 | 0.0214 | 0.1135 |
| **4** | 0.1621 | 0.6716 | 0.0182 | 0.0030 | 0.1165 |

| **Standardized Variance of the Mutation stats Explained by** | | | | | |
| --- | --- | --- | --- | --- | --- |
| **Canonical Variable Number** | **Their Own Canonical Variables** | | **Canonical R-Square** | **The Opposite Canonical Variables** | |
|  | **Proportion** | **Cumulative Proportion** |  | **Proportion** | **Cumulative Proportion** |
| **1** | 0.5497 | 0.5497 | 0.4775 | 0.2625 | 0.2625 |
| **2** | 0.2105 | 0.7602 | 0.2135 | 0.0449 | 0.3074 |
| **3** | 0.1615 | 0.9217 | 0.0809 | 0.0131 | 0.3205 |
| **4** | 0.0783 | 1.0000 | 0.0182 | 0.0014 | 0.3219 |

| Normal Quantile-Quantile Plot |
| --- |

The CANCORR Procedure

Canonical Redundancy Analysis

| **Squared Multiple Correlations Between the Network stats and the First M Canonical Variables of the Mutation stats** | | | | |
| --- | --- | --- | --- | --- |
| **M** | **1** | **2** | **3** | **4** |
| **BET** | 0.0011 | 0.0340 | 0.0398 | 0.0525 |
| **CLO** | 0.0226 | 0.0327 | 0.0976 | 0.0993 |
| **DEG** | 0.0370 | 0.0521 | 0.0695 | 0.0696 |
| **INDEG** | 0.0940 | 0.0941 | 0.0996 | 0.1013 |
| **OUTDEG** | 0.0046 | 0.0492 | 0.0747 | 0.0751 |
| **CORE** | 0.2728 | 0.2907 | 0.3001 | 0.3011 |

| **Squared Multiple Correlations Between the Mutation stats and the First M Canonical Variables of the Network stats** | | | | |
| --- | --- | --- | --- | --- |
| **M** | **1** | **2** | **3** | **4** |
| **log2(ΔM)** | 0.2927 | 0.3354 | 0.3355 | 0.3389 |
| **log2\|ΔM\|** | 0.2315 | 0.3380 | 0.3392 | 0.3392 |
| **log(h2M)** | 0.3805 | 0.3888 | 0.4019 | 0.4019 |
| **log(IM)** | 0.1452 | 0.1675 | 0.2054 | 0.2076 |
